# Extracellular Vesicles in the Cornea: Insights from Other Tissues

**DOI:** 10.1155/2021/9983900

**Published:** 2021-07-22

**Authors:** Tina B. McKay, Vincent Yeung, Audrey E. K. Hutcheon, Xiaoqing Guo, James D. Zieske, Joseph B. Ciolino

**Affiliations:** Department of Ophthalmology, Schepens Eye Research Institute of Mass Eye and Ear, Harvard Medical School, 20 Staniford Street, Boston, MA 02114, USA

## Abstract

Extracellular vesicles (EVs) are phospholipid bilayer-bound particles secreted by cells that have been found to be important in mediating cell-cell communication, signal transduction, and extracellular matrix remodeling. Their role in both physiological and pathological processes has been established in different tissues throughout the human body. The human cornea functions as a transparent and refractive barrier that protects the intraocular elements from the external environment. Injury, infection, or disease may cause the loss of corneal clarity by altering extracellular matrix organization within the stroma that may lead to detrimental effects on visual acuity. Over the years, numerous studies have identified many of the growth factors (e.g., transforming growth factor-*β*1, thrombospondin-1, and platelet-derived growth factor) important in corneal wound healing and scarring. However, the functional role of bound factors encapsulated in EVs in the context of corneal biology is less defined. In this review, we describe the discovery and characterization of EVs in the cornea. We focus on EV-matrix interactions, potential functions during corneal wound healing, and the bioactivity of mesenchymal stem cell-derived EVs. We also discuss the development of EVs as stable, drug-loaded therapeutics for ocular applications.

## 1. Introduction

Though their discovery dates back nearly three-quarters of a century, extracellular vesicles (EVs) have recently gained significant interest for their role in regulating physiological and pathological events important in human health and disease. An early study, published in 1946, first reported that specialized particles isolated from plasma following centrifugation possess diverse biological properties [1]. These sedimented particles were later described in 1967 as “platelet dust” that was clearly involved in blood coagulation [2]. It was in 1983 that Rose Johnstone's group applied immunogold labelling and transmission electron microscopy (TEM) to visualize the packaging of transferrin receptor into the multivesicular body and secretion of small EVs from a reticulocyte as it matured to a red blood cell [3, 4]. This foundational work, among others, paved the way in establishing EVs as distinct organelles that are secreted into the extracellular milieu in response to environmental changes and cell differentiation [5, 6]. This important finding, showing directed packaging of specific proteins into membrane-bound vesicles, sets the stage for later discoveries that defined fundamental roles for EVs in mediating cell-cell signaling, signal transduction, and extracellular matrix (ECM) remodeling in both physiological and pathological environments (reviewed by Yanez-Mo et al. [7]).

Since their discovery, EVs have since been classified into three broad subtypes based upon their biogenesis and size: exosomes (30-200 nm in diameter, packaged into the multivesicular body, and released by the endosomal pathway), microvesicles or ectosomes (100-1000 nm in diameter, arise from cell budding), and apoptotic bodies (0.5-2 *μ*m in diameter, result of cell compartmentalization during cell death) [8]. The methods for categorizing different EV subclasses have traditionally focused heavily on size, predominantly exosomes and microvesicles. Though this dichotomization has been challenged, it remains the main classification system for EVs, as the complexity regarding definitive protein markers and origin has not been clearly defined [9]. The International Society for Extracellular Vesicles endorses the term EVs, rather than exosomes or microvesicles, as the intracellular source and purity of the preparation are difficult to ascertain. General guidelines for using the term EVs are largely encouraged given the disparity in universal markers and the limitations in visualizing the formation of EVs in real time [10].

The molecular composition of EVs varies depending upon cellular origin and their biogenesis. EVs can incorporate a diverse repertoire of proteins, RNA, DNA, and lipids, which may lead to varied biological activity in the recipient cell. Characterization of EVs has been supported by the advancements of comprehensive databases (e.g., Vesiclepedia, ExoCarta, EVpedia, and exoRBase) that compile EV findings from numerous studies, with the aim of finding distinctive molecular signatures to specific cell/tissue types [11–13]. As a result, certain proteins, including classic exosomal markers, have been found to be present and may be used as EV markers: the tetraspanin proteins (CD9, CD63, and CD81), flotillin-1/-2, ESCRT-related genes (ALIX and TSG101), RABs, SNAREs, and others [14–16]. The larger microvesicles commonly share some protein markers that are found in exosomes, such as flotillin-1 and the major histocompatibility proteins (MHC-I and MHC-II), as well as enrichment of actinin-4 and mitofilin, among others [14]. Apoptotic markers, including Annexin V and C3b, have been identified on apoptotic bodies [17, 18]. EV markers independent of the cell of origin have also been reported, including ceramide enrichment, cholesterol, sphingomyelin, and other related lipids [19–21]. As the membrane of EVs is at least initially derived from the plasma membrane, EVs tend to retain the integrin markers of the cell of origin, which may contribute to delivering their bioactive cargo to specific cell types [22]. For corneal epithelial cells, these integrins include the *β*1 family of integrins (*α*2*β*1, *α*3*β*1, *α*6*β*1, *α*9*β*1, and *ανβ*1) [23, 24], with the *β*1 integrin subunit found in EVs secreted by these cells [25].

EVs have been detected in numerous biological fluids, including blood, lymph, saliva, urine, sweat, and tears [26–31], and are generally conserved throughout the animal kingdom from humans to microorganisms [32]. EVs are capable of mediating long-distance endocrine signaling, in addition to local paracrine or autocrine signals, depending on whether the EVs are endocytosed or secreted. For example, EVs released into circulation from adipose tissue have been shown to contain miRNAs that regulate gene and protein expression of metabolic factors within the liver and influence energy stores and glucose tolerance [33, 34]. Secretion of EVs during cardiovascular exercise likewise modulates liver function and may stimulate energy expenditure and metabolism [35]. In terms of pathology, EVs secreted from tumor cells have been found to be involved in the development, progression, and metastasis of certain cancers by actively transporting chemotherapeutics out of the cell [36] and priming a tumor-supportive microenvironment (reviewed by Shephard et al. and Webber et al. [37, 38]). The presence of oncogenes and onco-miRNAs within EVs may also confer resistance to select chemotherapeutics. Serum samples from HER2-positive breast cancer patients have been found to possess an abundance of EVs expressing HER2, a gene associated with promoting aggressive and metastatic cancer [39]. EV-mediated resistance to the antibody-based drug, trastuzumab, which selectively targets the overexpressed HER2 receptor has been reported [40]. These studies highlight the importance of EVs in short- and long-range cellular communication within the body and set the stage for a growing interest in EVs in human physiology.

## 2. Early Evidence of EVs in the Cornea

The cornea is composed of three major cellular layers—the corneal epithelium, stroma, and endothelium—and two acellular layers, Bowman's layer and Descemet's membrane, at the anterior and posterior regions, respectively. The bulk weight and volume of the cornea are made up by the hydrated stroma where resident keratocytes, immune cells, and nerve fiber bundles are dispersed in a highly organized ECM composed primarily by collagen types I and V. Blunt trauma, infection, chemical injury, and ocular or systemic disease may cause changes in ECM organization leading to corneal haze or scar development and visual impairment.

**Figure 1:**
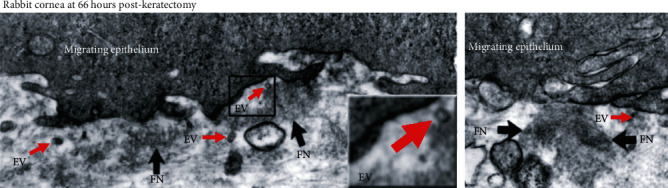
Transmission electron microscopy (TEM) images of a rabbit cornea 3 days postkeratectomy. The presence of EVs (*red arrow*s) and fibronectin (FN, *black arrows*) is seen within the anterior stroma near the migrating epithelium. Original magnification: 31,200x. Inset magnification: 2.5x. Images were modified and reproduced from Zieske et al. 1987 [41] with permission from Dr. James D. Zieske (author), ARVO (copyright holder).

Dr. James D. Zieske, along with Dr. Ilene Gipson, published a notable paper in 1987 identifying the abundant expression of fibronectin following injury, which accounted for roughly 2% of the total protein within the wounded anterior stroma postkeratectomy [41]. In organ cultures, the source of the fibronectin was attributed to activated keratocytes since corneal epithelial cells appeared to express very little [41]. This data is consistent with studies showing increased fibronectin transcript levels by fibroblasts following injury [42]. This early study also described the appearance of membrane-bound particles within the anterior stroma by 3 days following a keratectomy, thus providing one of the first evidences for the presence of EVs in the cornea during wound healing (Figure 1). The use of the broad term “polysomes” in this landmark paper specified the diversity of membrane-bound microparticles observed in the wounded area in terms of cellular origin, cargo, and functionality that collectively may have very different effects depending on the subclass. More recently, we have found that human corneal epithelial cells express both fibronectin and thrombospondin-1 when cultured *in vitro*, with significant amounts also present in secreted EVs [25]. These EVs have been proposed to serve as a targeted source of fibronectin to the epithelial-stromal interface following wounding [43], though direct evidence of the release of bound provisional matrix proteins from EVs has not been shown to date.

In terms of marker expression, while the corneal epithelium shows high expression of the tight junctional protein, ZO-1, vesicles found within the epithelium do not appear to contain ZO-1, as assessed by immunogold TEM [44]. Similar to other cell types, EVs isolated from corneal epithelial cells have been found to contain the tetraspanin proteins, CD63 and CD9, as well as select laminin subunits (e.g., laminin *α*-3, *α*-4, *β*-1, *γ*-1, and *γ*-2) [25].

The presence of EVs secreted from the wounded epithelium has also been observed during basement membrane reformation. While a keratectomy removes the overlaying epithelium and basement membrane, along with a portion of the anterior stroma, a mild thermal burn to the ocular surface also leads to cell-mediated dissolution of the basement membrane via controlled release of matrix metalloproteinases [45, 46]. The migrating epithelium then deposits a fresh basement membrane, during which the formation of “blebs” become apparent at the basal edge of the epithelial cells [45]. These structures are the result of outward budding of portions of the plasma membrane that form microvesicles that may release bound cytoplasmic content into the extracellular milieu. Whether secretion of these EVs from the epithelium mediates reformation of the basement membrane is not clear; however, key basement membrane and provisional matrix proteins have been reported in EVs derived from corneal epithelial cells cultured *in vitro* [25].

## 3. EVs and Matrix Interactions

Current understanding regarding the biosynthesis of collagen in the cornea focuses on procollagen cleavage, lamellar organization, and ultimately, fibril cross-linking that leads to an ECM of sufficient stiffness to withstand external pressure, yet malleable and transparent to allow for vision (reviewed by McKay et al. and Meek and Knupp [47, 48]). The corneal stroma is composed of collagen fibrils with a relatively uniform diameter of ~25 nm. Collagen type V is known to serve a fundamental role in regulating collagen fibril diameter in the cornea by limiting the number of collagen type I monomers that may bind [49, 50]. Interactions between collagen and proteoglycans, such as lumican and decorin, also influence collagen fibrillogenesis and fibril diameter [51, 52]. These small collagen fibrils found in the cornea are thought to be required to permit complete tissue transparency and enable proper corneal curvature, elasticity, and rigidity.

**Figure 2:**
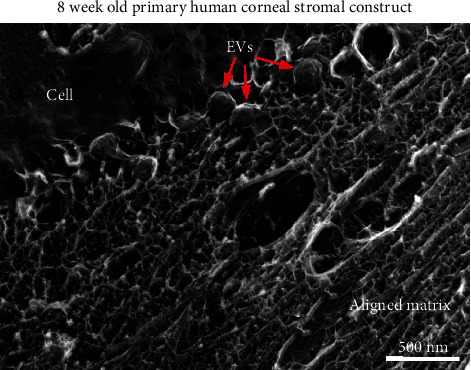
EVs (*red arrows*) budding from corneal fibroblasts (*cell*) in a self-assembled collagen matrix (*aligned matrix*). Image was modified and reproduced from Ren et al. 2008 [53] with permissions. Copyright (2008) Wiley-Liss, Inc.

Collagen fibrils, proteoglycans, and other ECM proteins play a concerted effort in regulating the binding and activation of secreted growth factors and, likewise, may influence EV migration and cell uptake. Studies in tissue-engineered corneal models have shown the presence of EVs distributed within the collagen matrix [43]. The presence of EVs secreted by human corneal fibroblasts on the surface of a self-assembled ECM has been observed by TEM with maintenance of the characteristic rounded morphology (Figure 2).

**Figure 3:**
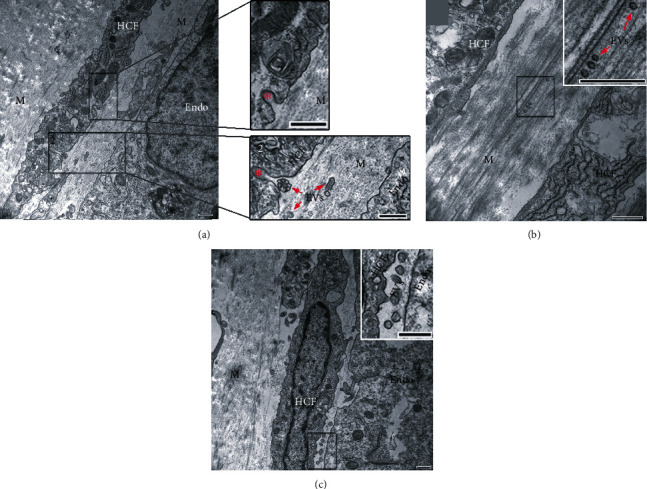
EVs distributed in a 3D tissue-engineered corneal endothelial-stromal model. (a) A coculture of human corneal fibroblast (HCF) and human corneal endothelial cells (Endo) shows (1) endocytosis/exocytosis (*red asterisks*) and (2) EVs (*red arrows*) present in the matrix (M) and a cluster of EV endocytosing/exocytosing. (b) EVs distributed within the stromal matrix. (c) EVs present between an HCF and Endo cell. Bars = 500 nm. Images were modified and reproduced from Zieske et al. (2020) [56] based on a Creative Commons BY license (CC BY 4.0), doi:10.1002/ar.24181.

Secretion and/or uptake of EVs have also been observed in a tissue-engineered model of the corneal stroma and corneal endothelium (Figure 3). This coculture model is constructed using human corneal fibroblasts that have secreted and assembled a collagen-rich matrix over a time period of one month followed by seeding of human corneal endothelial cells onto the mature stromal construct [54]. This model recapitulates corneal stromal-endothelial cell interactions found in the corneal tissue *in vivo* with a distribution of a self-assembled ECM and distal cell interactions [55]. The presence of EVs can be visualized within the matrix and localized between cell types supporting the application of these sophisticated tissue models in the study of EV-mediated cell-cell communication.

**Figure 4:**
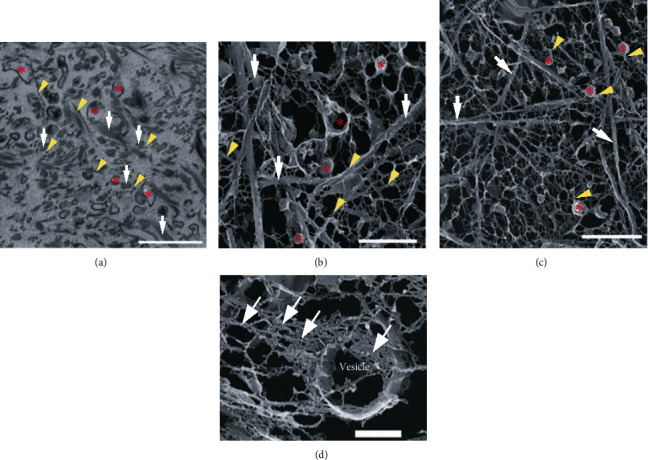
EV-collagen interactions in a reconstituted collagen substrate. (a) TEM image of EVs (*red asterisks*) distributed within the collagen matrix. A number of large collagen aggregates are attributed to PureCol collagen (*white arrows*) with the presence of small fibrils (*yellow arrowheads*). Scale bar = 1 *μ*m. (b) High-magnification QFDE image shows EVs (*red asterisks*), large fibril bundles (*white arrows*), and small fibril bundles (*yellow arrowheads*) present within the collagen matrix. Scale bar = 1 *μ*m. (c) Low-magnification QFDE image shows EVs (*red asterisks*) connected with small fibril bundles (*yellow arrowheads*). Large fibril bundles (*white arrows*) were also present. Scale bar = 2 *μ*m. (d) High-magnification QFDE image of an EV (*vesicle*) containing matrix components (*white arrows*). Scale bar = 0.5 *μ*m. Images were modified and reproduced from Saeidi et al. 2012 [57] based on a Creative Commons license, doi:10.1002/bit.24533.

The use of quick-freeze/deep-etch (QFDE) electron microscopy has provided a unique method to evaluate collagen organization and EV interactions with collagen fibrils (Figure 4). An abundance of large and small EVs, presumably secreted by distant corneal fibroblasts, is distributed throughout the collagen matrix (Figures 4(a)–4(c)). The presence of polymerized fibrils emanating from a secreted EV provides supporting evidence that EVs may be a conduit for release of ECM components (Figure 4(d)). These findings are consistent with data from our lab showing an abundance of provisional matrix proteins, including fibronectin and thrombospondin-1, as well as basement membrane proteins, laminin and collagen type IV, present in corneal epithelial cell-derived EVs [25]. However, little is known regarding the mechanisms involved in the release of EV-bound proteins into the extracellular space.

Evidence in hard tissues, such as bone and cartilage, has provided clues to a potential role for EVs in mediating matrix deposition. Bone mineralization involves integration and deposition of minerals (e.g., calcium and phosphate ions) in the space between collagen type I fibrils, thereby forming a stiffened matrix to provide structural support for softer tissues. The presence of EVs rich in the catalytic enzyme, alkaline phosphatase, has been observed in bone and regions of cartilage growth [58–61]. Alkaline phosphatase is expressed in a number of tissues in the human body, including the bone, liver, and intestine, and functions as an enzyme for phosphate monoester substrates to generate an alcohol and monophosphate ion. One of the major components in hydroxyapatite crystals is inorganic phosphate, which forms a complex with calcium. The following different mechanisms have been proposed for the functional role of these EVs in mediating calcification: regulation of phosphate levels, promotion of apatite crystal formation, and binding interactions with the collagen and proteoglycans in bone or cartilage [62]. These EVs are highly electron dense and form hydroxyapatite that may serve as a nucleation site for matrix deposition [63]. However, whether this phenomenon occurs during ECM formation and/or remodeling in soft tissues, such as the cornea, remains unclear.

## 4. EVs and Wound Healing

**Figure 5:**
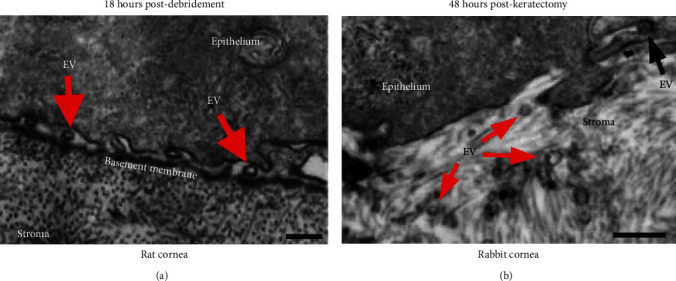
TEM images of the epithelial-stromal interface in the corneas with varying severity of wounds: (a) debridement (basement membrane left intact) and (b) keratectomy (basement membrane removed). (a) Localization of EVs (*red arrows*) on the anterior side of the epithelial basement membrane in a rat cornea at 18 hours postdebridement. Scale bar = 250 nm. (b) Dispersion of EVs (*red arrows*) in the anterior stroma in a rabbit cornea at 48 hours postkeratectomy. EV undergoing endocytosis/exocytosis (*black arrow*). Scale bar = 250 nm. Images were modified and reproduced from Han et al. 2017 [64] based on a Creative Commons license (CC BY 4.0), doi:10.1038/srep40548.

EVs have been found to be secreted in the cornea following wounding [41, 64, 65]. In an epithelial debridement model, numerous EVs were shown to be present at the basal side of the migrating epithelium and apical to the basement membrane (Figure 5(a)). Interestingly, the epithelial basement membrane appeared to limit the diffusion of these epithelial EVs to the stroma [56, 64] in a manner similar to that observed with growth factors found in the tear film. These growth factors, such as transforming growth factor-beta1 (TGF-*β*1), typically correlate with corneal scarring following injury where the basement membrane is damaged [65, 66]. As seen in Figure 5(b), when the basement membrane is removed by keratectomy, EVs appear to pass into the stroma and potentially communicate with the stromal cells or matrix. For TGF-*β*1 and related growth factors, binding of the TGF-*β* prodomain may occur with basement proteins, such as perlecan and nidogen-1, mediated via electrostatic interactions and chemical association heparin sulfate proteoglycans [67]. These interactions have been observed in *in vitro* systems using binding assays to assess protein affinity in a static or microfluidic environment. Restricted permeability of the corneal epithelial basement membrane to TGF-*β*1 has been purported as a protective measure to prevent corneal scarring [66, 68]. A similar binding interaction involving epithelial cell-derived EVs and the epithelial basement membrane may also explain the resistance to scarring in debridement models when the basement membrane remains intact compared to following a keratectomy, in which the basement membrane is removed, and scar development is more common.

Similar to the fibrillar material contained within EVs shown in a tissue-engineered stromal system [57], secreted EVs may also contain basement membrane proteins. We have found that isolated EVs secreted by a human corneal epithelial cell line promote myofibroblast differentiation when applied to corneal fibroblasts cultured in a 3D *in vitro* stromal model [25]. Protein analysis of isolated corneal epithelial cell-derived EVs has identified proteins associated with provisional matrix, such as thrombospondin-1 and fibronectin, suggesting that EVs may contain proteins associated with basement membrane reformation [25]. We have found that EVs isolated from human corneal endothelial cells also contain basement membrane proteins, including laminin and heparan sulfate proteoglycan core proteins (*unpublished data*), suggesting that EVs may also be conduits for transfer and assembly of the basement membrane from neighbouring cells. It could be hypothesized that EV-associated heparan sulfate proteoglycans are responsible for EV internalization into their targeted recipient cells and contribute to their functional activity [69]. These studies provide evidence that the deposition of a proper corneal epithelial basement membrane, at least *in vitro*, requires the presence of stromal and/or corneal endothelial cells [70]. Biochemical analyses have shown that both corneal keratocytes and fibroblasts express epithelial basement membrane proteins, such as perlecan and nidogen-2, *in vitro* [71], suggesting that corneal stromal fibroblasts cells may serve as a key source of basement membrane proteins following epithelial injury to accelerate basement membrane reformation. Notable questions remain, however, regarding the role of EVs as stable carriers of basement membrane proteins and the mechanism(s) by which these rather large proteins escape the bilayer membrane of EVs.

## 5. Mesenchymal Stem Cell-Derived EVs

Numerous studies have described the antifibrotic properties of mesenchymal stem cell- (MSC-) derived EVs in the cornea and skin [72]. The antiscarring properties of MSCs have been at least partially attributed to microRNAs encapsulated within EVs. Of particular interest, the immunomodulatory properties of MSC-derived EVs suggest that these vesicles may be useful in improving patient-centered outcomes following corneal transplantation.

Our current understanding of MSCs isolated from bone marrow [73] and other adult tissues (e.g., adipose, Wharton's jelly, and cornea) [74–76] is that they have the capacity to differentiate into mesoderm-derived lineages that possess regenerative, reparative, and immunomodulatory properties. By meeting the minimum framework of human MSCs as defined by the International Society for Cellular Therapy (ISCT) [77], the therapeutic application of MSCs has been highlighted to be effective on a wide range of animal models, to reduce corneal scarring [78, 79], restore corneal transparency [80], and exert corneal antifibrotic effects [81, 82].

Indeed, the early reports of MSC multipotent differentiation capacity fuelled the initial enthusiasm for the new regenerative paradigm by donor cell engraftment. Subsequent studies, however, clarified that paracrine factors play a huge role, in the mechanism of MSC therapeutic action, verified in many independent studies targeting a variety of different tissues, including the kidney, heart, nervous tissues, skeletal muscle, eyes, lung, and placenta [83–91].

With the focus of administering MSC-derived EVs on different corneal disease models, it was reported that topical application of corneal MSC-derived EVs accelerated corneal epithelial wound healing [92], decreased corneal epithelial defects, and reduced inflammatory cytokine production in mice with desiccating stress [93]. Significantly, and congruently, the enhanced proliferation and suppressed apoptosis, as well as the suppressed proinflammatory properties in corneal epithelial cells treated with MSC-derived EVs, confer a similar reparative effect on corneal wound repair [94] as induced pluripotent stem cell-derived EVs [95]. In addition, MSC-derived EVs were observed to alter corneal stromal cells by promoting ECM synthesis, changing matrix metalloproteinases and collagen levels, and increasing stromal cell proliferation [96]. A collective effort remains in deciphering the therapeutic mechanism of MSC-derived EVs. One study reported that corneal stem cell-derived EVs lacking unique miRNA sets (miR-23-3p, miR-191-3p, miR-221-39, and miR-222-3p) were ineffective in reducing inflammation and blocking corneal scarring after wounding [97]. This strongly suggests that MSC-derived EV treatment for corneal-related injuries may prove to be efficacious in restoring homeostasis at the onset of injury.

## 6. Drug-Loaded EVs as Therapeutics

Within the past decade, EVs have emerged as an attractive candidate for the new generation of a natural nanoscale delivery system. Attributed to their intrinsic ability to internalize an array of antigens to elicit a biological response to target cells, a multitude of studies have focused on exploiting EVs as therapeutic carriers. These EVs are ideal nanoscale carriers for potential clinical applications owing to their capacity to avoid rapid clearance by the mononuclear phagocyte system [98], overcoming immunotoxicity [99], and staying in the body's circulation longer due to the negative zeta potential or deeper penetration into tissues due to their size [100].

The main approaches for loading therapeutic cargo (e.g., functional RNA, DNA molecules, peptides, and synthetic drugs) include active and passive encapsulation. The active cargo approach involves the temporary disruption of the EV plasma membrane by sonication or electroporation [101]. In contrast, the passive method uses diffusion, where drugs (e.g., paclitaxel and curcumin) load along the concentration gradient, depending on their hydrophobicity [102]. Many studies predominantly focused on loading therapeutics into EVs in the field of oncology. This was initially reported with loading curcumin into EVs derived from various cell types (e.g., mouse embryonic fibroblasts, mouse lymphoma, and human adenocarcinoma cells), which showed better solubility and anti-inflammatory bioactivity compared to traditional curcumin administration [103, 104]. This study paralleled those demonstrating that loading paclitaxel into MSCs [105], LNCaP or PC-3 prostate cells [106], or taxol into MSCs [107] resulted in enhanced cytotoxicity and inhibition of tumor cell growth. There have been numerous studies [101] showcasing chemotherapeutic drug loading into EVs, showing higher efficacy and superior bioavailability, but research has been sparse regarding ocular pharmacology.

One study reported that EV-associated adenoassociated virus type 2 (EV-AAV-2) from 293 T cells demonstrated deeper penetration via intravitreal injection in the retina, efficiently reaching the inner and outer plexiform compared to conventional AAV-2, thereby suggesting that this treatment may be an effective method for intravitreal gene transfer into the retina [108]. Furthermore, another study showed that MSC-EVs overexpressing miR-126 successfully suppressed the HMGB1 signaling pathway and suppressed hyperglycemia-induced retinal inflammation in rats [109]. So far, however, there has been limited research on loading therapeutic cargo onto EVs to treat eye-related diseases or injury. Therefore, further endeavours are required to develop novel therapies in ophthalmology.

## 7. Conclusions

The laboratory of Dr. James D. Zieske, and many others, has made pivotal discoveries over the years investigating the fundamental mechanisms involved in corneal wound healing. This work includes the initial discovery of EVs in the wounded cornea in 1987, with the observation of EV-collagen interactions nearly twenty years later, and finally, the proteomic and functional characterizations of epithelial cell-derived EVs in 2020. The development and application of biologically relevant tissue-engineered models of the human cornea, including 3D self-assembled stromal models and epithelial- and endothelial-stromal cocultures, provided further evidence regarding the key players that may promote corneal scarring (e.g., TGF-*β*1, platelet-derived growth factor, and epithelial cell-derived EVs) and those that may inhibit scarring (e.g., TGF-*β*3 and mitomycin C). The growing interest in EVs by the Zieske group through the years may perhaps be linked to their innovative and early use of high-resolution imaging (TEM and confocal microscopy) to visualize changes in corneal tissue structure and protein expression in different animal and tissue models, a constant theme from this group that helped define the temporal cascade involved in corneal wound healing. The discovery of the presence of these submicron-, phospholipid bilayer membrane-bound particles in the cornea occurred with the visualization of corneal tissue sections by electron microscopy, in a similar manner to how they were originally discovered in reticulocytes around the same time. EVs secreted from the corneal epithelium in response to wounding were eventually identified and appear to be localized to the anterior basement membrane following debridement, but not following a keratectomy. Largely absent in the uninjured cornea, the abundance of EVs in the anterior cornea following superficial wounding prompted the supposition that EVs secreted by the corneal epithelium in response to tissue damage may have an important function during corneal repair.

**Figure 6:**
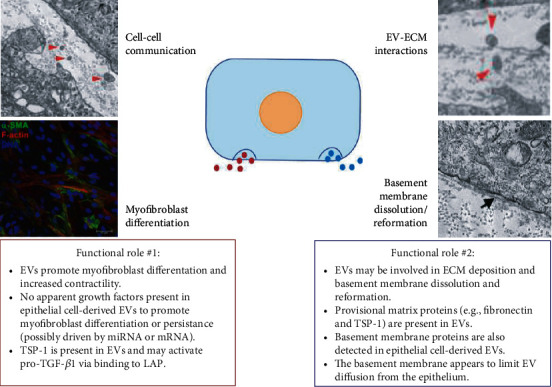
Proposed functional roles of EVs in the cornea. The presence of EVs (*red arrowheads*) dispersed in the ECM of a self-assembled corneal fibroblast construct. A basement membrane (*black arrow*) forms between corneal epithelial cells and the stromal matrix. Images were modified and reproduced from McKay et al. (2019 and 2020) [25, 43] based on a Creative Commons BY license (CC BY 4.0), doi:10.3390/cells9051080 and doi:10.3390/bioengineering6040110.

Consistent with the diverse properties of EVs in other tissues throughout the body, EVs derived from—and secreted within—the cornea also appear to mediate various functional outcomes that may influence cell phenotype (e.g., myofibroblast differentiation), ECM structure (e.g., matrix contraction and fibrotic composition), and epithelial-stromal interactions (e.g., basement membrane dissolution and reformation) (Figure 6). It is likely that different subpopulations of corneal EVs have diverse effects depending on the cell of origin, relative abundance, and the target cell. Parsing out these different functional roles of EVs in the cornea will require careful isolation and biochemical analyses to define reproducible surface markers consistent with specific EV subpopulations and their associated properties (i.e., EVs that promote myofibroblast differentiation compared to EVs that carry provisional matrix proteins). Likewise, identifying how wounding or disease influence EV composition and secretion may help in understanding corneal tissue regeneration and fibrosis and the underlying mechanisms that determine the clinical outcome (e.g., scarless healing or scar development). The antifibrotic and immunomodulatory properties of MSC-derived EVs provide solid evidence that isolated EVs may serve as a targeted therapeutic approach to promote corneal wound healing. Clearly, further studies are needed to provide mechanistic insight into the role of EVs in the cornea in the context of wound healing and disease. This work is technically challenging, but no doubt will help advance our understanding of cell communication in the cornea and the role of the microenvironment in tissue regeneration.

## Data Availability

Data sharing is not applicable to this article as no datasets were generated or analysed during the current study.
